# Optimization of an *ex vivo* gene transfer to the hamstrings tendons muscle remnants: potential for genetic enhancement of bone healing

**DOI:** 10.3325/cmj.2019.60.201

**Published:** 2019-06

**Authors:** Eduard Rod, Igor Matić, Maja Antunović, Vesna Vetma, Ivan Pavičić, Damir Hudetz, Inga Marijanović, Dragan Primorac, Alan Ivković

**Affiliations:** 1 St. Catherine Specialty Hospital, Zabok/Zagreb, Croatia; 2 Department of Molecular Biology, Faculty of Science, University of Zagreb, Zagreb, Croatia; 3 Institute for Medical Research and Occupational Health, Croatia; 4 Eberly College of Science, The Pennsylvania State University, University Park, PA, USA; 5 School of Medicine, University of Split, Split, Croatia; 6 J. J. Strossmayer University of Osijek, School of Medicine, Osijek, Croatia; 7 Institute for Anthropological Research, University of Zagreb, Zagreb, Croatia; 8 Faculty of Medicine, University of Rijeka, Rijeka, Croatia; 9 Henry C. Lee College of Criminal Justice and Forensic Sciences, University of New Haven, West Haven, CT, USA; 10 Department for Orthopedic Surgery, University Hospital “Sveti Duh,” Zagreb, Croatia; 11 Department of Biotechnology, University of Rijeka, Rijeka, Croatia; 12 Department of Histology and Embryology, University of Zagreb School of Medicine, Zagreb, Croatia

## Abstract

**Aim:**

To assess whether an adenoviral vector carrying the bone morphogenetic protein genes (Ad.BMP-2) can transduce human muscle tissue and direct it toward osteogenic differentiation within one hour.

**Methods:**

This *in vitro* study, performed at the Department of Molecular Biology, Faculty of Science, Zagreb from 2012 to 2017, used human muscle tissue samples collected during anterior cruciate ligament reconstructions performed in St Catherine Hospital, Zabok. Samples from 28 patients were transduced with adenoviral vector carrying firefly luciferase cDNA (Ad.luc) by using different doses and times of transduction, and with addition of positive ions for transduction enhancement. The optimized protocol was further tested on muscle samples from three new patients, which were transduced with Ad.BMP-2. Released bone morphogenetic protein 2 (BMP-2) levels in osteogenic medium were measured every three days during a period of 21 days. Expression of osteogenic markers was measured at day 14 and 21. After 21 days of cultivation, muscle tissue was immunohistochemically stained for collagen type I detection (COL-I).

**Results:**

The new transduction protocol was established using 10^8^ plaque-forming units (*P* < 0.001) as an optimal dose of adenoviral vector and 30 minutes (*P* < 0.001) as an optimal contact time. Positive ions did not enhance transduction. Samples transduced with Ad.BMP-2 according to the optimized protocol showed enhanced expression of osteogenic markers (*P* < 0.050), BMP-2 (*P* < 0.001), and COL I.

**Conclusion:**

This study confirms that Ad.BMP-2 can transduce human muscle tissue and direct it toward osteogenic differentiation within 30 minutes.

Regenerative medicine looks for the ways to stimulate the inherent ability of our body to regenerate damaged tissue by activating or promoting natural healing processes, thus lowering the number of needed surgical or other medical interventions. It focuses on the interaction of cells, biological signals, and the environment (1). One of the most important topics in orthopedics is bone regeneration. Essential components needed for bone regeneration are osteogenic cells, osteoinductive growth factors, and an osteoconductive structure in the local environment (2,3). Various human cells have the potential to differentiate toward osteolineage cells. For example, bone-marrow derived mesenchymal stem cells (4) and stem cells derived from anterior cruciate ligament (ACL) remnant have been used to promote bone healing (5). Moreover, the insights into the creation of heterotopic bone (6) and the disease fibrodysplasia ossificans progressiva (7) have revealed a significant ability of human muscles to create bone. Muscle tissue contains various populations of progenitor cells: satellite, non-satellite, and perivascular cells. Non-satellite mesenchymal progenitors and perivascular cells play the main role in heterotopic bone formation (8).

Although human muscle-derived cells can enhance bone regeneration (9), they often lack sufficient biological stimuli by growth factors, the most potent of which are bone morphogenetic proteins (BMP) -2, -4, and -7 (10-12). Successful osteogenesis depends on appropriate spatial and temporal expression of BMPs. BMP-2 has been used to promote bone regeneration (4,5,9), but it is soluble and disappears soon after implantation, so that many researchers have sought the delivery system to retain BMP-2 at the target site. This search led to the BMP-2-expressing viral vectors, as a concept representing gene therapy. The goal of gene therapy is to implement genetic material into cells, thus providing sufficient concentration of the missing substrate and alleviating the disease symptoms (13). This new genetic material is transferred to cells using virus vectors carrying genes needed for treatment.

In recent years, many of these novel techniques have been developed for the treatment of musculoskeletal diseases, both for healing of bone and soft tissues (1,14-16). However, these methods have their limitations and need to be further improved. An example of this kind of treatment is the conventional *ex vivo* gene therapy, which was the initial modality of gene therapy and the first one used in clinical trials on humans (17). It consists of several steps: harvesting of cells from the body, *in vitro* cultivation, infection of cells with virus vectors, and replantation of genetically modified cells back to the donor (13). Such a strategy has been proven successful, although not ideal for application during orthopedic procedures. Tissue transportation and cultivation in a laboratory requires two-steps surgical procedures, making the whole process expensive, long lasting, and inconvenient for the patient. To avoid such a complicated procedure, the expedited *ex vivo* strategies have been developed. The idea behind them is to perform a successful gene therapy inside the operating theater during a single procedure and without the need for extensive tissue cultivation. Several studies of gene therapy used for bone and cartilage healing proved this to be possible, but the whole process lasted minimally two hours (4,18). Although this is a significant step forward compared with the conventional *ex vivo* gene therapy, the average duration of orthopedic procedures is often shorter than two hours. Consequently, these strategies cannot be clinically implemented in some of the most common orthopedic procedures, such as ACL reconstruction, and need optimization. The efficiency of gene therapy can be improved by enhancing the cell transduction with adenoviral vectors through prevention of electrostatic rejection between the negative ions of cell surfaces and adenoviruses (19-21), development of new serotypes of adenoviruses (22), and changing virus envelope proteins to improve tissue specificity and cell surface binding (23). Owing to the binding ability between negative cell surface charge and adenovirus vectors, positive La^3+^ increase the percentage of transduced cells and the transgenic expression per cell (21).

The aim of our study was to create a model of gene therapy for bone regeneration that could be performed as a one-step procedure, with abundant and easily harvested tissue, and the surgery duration in reasonable boundaries, thus lowering the chances of complications. We hypothesized that Ad.BMP-2 was capable to transduce human muscle tissue and direct it toward osteogenic differentiation within a period shorter than one hour.

## MATERIALS AND METHODS

### Study design

This single center *in vitro* analytic study was performed at the Department of Molecular Biology, Faculty of Science, University of Zagreb from 2012 to 2017. The study was approved by the Ethics Committee of St. Catherine’s Hospital on November 7, 2012 (07112012). The muscle samples were collected during ACL reconstructions performed in St Catherine Hospital, Zabok from 2012 to 2016. The study involved 31 patients (between 18 and 55 years) who previously completed informed consent forms for joining the study and data publication. We divided our study into four phases. In the first three phases we used Ad.Luc as a standard for measuring viral transduction of muscle cells. In the first phase we estimated the optimal dose of Ad.Luc for human muscle tissue transduction. In the second phase we determined the optimal time in which this dose of Ad.Luc should be in contact with the human muscle tissue. The third phase included testing whether different concentrations of La^3+^ and Ca^2+^ cations in combination with the optimal dose and time affect viral transduction of the cells. In the fourth phase, we used Ad.BMP-2, following an optimized protocol from the first three phases. We measured the gene expression of *BMP-2* every 3 days during a period of 21 days and osteogenic markers *runt-related transcription factor* (*RUNX2*), *bone sialoprotein* (*BSP*), and *dentin matrix protein 1* (*DMP-1*) on day 14 and 21. We also immunohistochemically detected collagen type I (COL-I) on day 21.

### Tissue samples

The muscle samples were collected during ACL reconstructions performed in St Catherine Hospital. Muscle tissue was removed from the tendon muscular part and transported into Dulbecco's Modified Eagle Medium (DMEM)-high glucose (Lonza, Basel, Switzerland) supplemented with 10% fetal bovine serum (FBS) (Gibco Laboratories, Gaithersburg, MD, USA) and 1% penicillin (pen) and streptomycin (strep) (Invitrogen, Carlsbad, CA, USA).

### Vectors

We used first generation adenoviruses (ΔE1, ΔE3), serotype 5, carrying human BMP-2 (Ad.BMP-2) and firefly luciferase cDNA (Ad.Luc) under the transcriptional control of the human cytomegalovirus (24). Adenoviruses were kindly donated by Professor Christopher H. Evans (Harvard Medical School, Boston, MA, USA). Vectors were propagated in HEK-293 cells and further purified using cesium chloride density gradient, followed by dialysis. Viral stocks were stored in 10% glycerol at −80°C. The viral particles concentration was measured by optical density measurement (Ad.BMP-2, 2.42 × 10^12^ viral particles/mL ±0.25; Ad.Luc, 1.42 × 10^12^ viral particles/mL ±0.32), while the number of infective viral particles was estimated by virus plaque assay (Ad.BMP-2, 7.6 × 10^9^ PFU/mL; Ad.Luc, 6.1 × 10^9^) (25).

### Phase 1: determination of optimal viral dose

Muscle tissue (8 patients) was cut into pieces (mass 50 μg) and transferred into 500 μL of DMEM into 24 well plates. The medium was removed and the samples transduced with 0, 10^5^, 10^6^, 10^7^, and 10^8^ plaque-forming units (PFU). 20 μL of appropriate viral dilution was dropped directly on the samples and incubated for 1 h at 37°C supplemented with 5% CO_2._ Following incubation, 1 mL of DMEM was added to each sample and returned to the incubator for another hour. Finally, DMEM was aspirated, and the samples were washed three times in 2 mL of phosphate-buffered saline (PBS). Furthermore, 1 mL of fresh medium was added, and 72 h later total proteins were isolated and luciferase assay performed. For positive control, 10^8^ PFU (20 μL) was directly added onto the muscle sample, the tissue was not washed after 1 h in PBS, only DMEM was added. The tissue samples stayed in contact with the virus until the 72 h endpoint.

### Phase 2: determination of the optimal contact time

Muscle tissue (10 patients) was cut and processed as previously described, followed by transduction with previously determined optimal viral dose. The samples were incubated for 0, 15, 30, 60 min, and 72 h at 37°C supplemented with 5% CO_2_. Following the incubation period, the samples were processed in an identical manner as previously described and, after 72 h total proteins were isolated and luciferase assay was performed.

### Phase 3: the effect of lanthanum and calcium ions on transduction

Muscle tissue (10 patients) was transduced with the previously determined optimal dose and contact time, with the addition of ions as follows: Ad.Luc; Ad.Luc +0.2 mM La^3+^; Ad.Luc +5 mM Ca^2+^; and Ad.Luc +0.2 mM La^3+^ + 5 mM Ca^2+^. The samples were washed in PBS and incubated for 72 h in an incubator, followed by protein isolation and luciferase assay.

### Total protein extraction and luciferase assay

Total protein extracts were obtained using CelLytic M (Sigma-Aldrich, Taufkirchen, Germany). Briefly, DMEM was aspirated; the samples were washed three times in 2 mL of PBS, frozen in liquid nitrogen, and homogenized with a mortar and pestle. 300 μL of CelLytic M was added to each sample. Protein concentration was determined using the bicinchoninic acid protein assay kit (Thermo Fisher Scientific, Waltham, MA, USA) according to manufacturer's instructions. In Opaque 96-Well Microplate, 100 μL of total protein extract was mixed with 100 μL of luciferase assay buffer (1 mM adenosine triphosphate, 0.25 mM luciferin, 1% bovine serum albumin, 8 mM MgCl_2_, 0.4 mM dithiothreitol, 1 mM ethylene glycol-bis(β-aminoethyl ether)-N,N,N′,N′-tetraacetic acid) and luciferase enzyme. Luminescence was measured by using microplate reader (Victor Multilabel X, Perkin Elmer, Waltham, MA, USA).

### Phase 4: the application of optimized transduction protocols for enhanced osteodifferentiation of muscle tissue using AD.BMP 2

Every muscle tissue (3 patients) was cut into 4 × 50 μg pieces and separated into 2 groups (two samples in one group) to evaluate its osteogenic response to transduction with Ad.BMP-2. One group of doubled samples was transduced with Ad.BMP 2 viral vector by applying the previously optimized protocol and the other group of doubled samples was not transduced. All samples were thoroughly washed in PBS and incubated in osteogenic medium (Minimum Essential Medium – Alpha Eagle, 10% FBS, 1% pen/strep, 50 μg/mL ascorbic acid, and 10 mM β-glycerophosphate) over a period of 14 and 21 days.

The osteogenic media were replaced every 3 days, and released BMP-2 levels were measured with an ELISA kit (R&D Systems, Minneapolis, MN, USA) according to the manufacturer's protocol. The expression of osteogenic markers *RUNX2*, *BSP,* and *DMP1* was measured by real-time quantitative PCR (qPCR) at day 14 and 21. After 21 days of cultivation, muscle tissue was prepared for immunohistochemical staining and COL-I detection.

### Gene expression studies

Total RNA (2 μg) was isolated using TRIzol reagent (Invitrogen) according to manufacturer's instructions following DNase I treatment (New England Biolabs, Ipswich, MA, USA). cDNA was synthetized under the following conditions: 10 min at room temperature, 1 h at 42°C, 5 min at 99°C, and 5 min at 5°C. The expression of *RUNX2* (Mm00501584_ m1), *DMP-1* (Ms01009391_g1), and *BSP* (Hs00173720_m1) on days 14 and 21 was analyzed with TaqMan® Gene Expression Assays (Thermo Fisher Scientific). QPCR was performed on the 7500 Fast PCR system (Applied Biosystems) under the following conditions: 10 min at 95°C for 1 cycle, 15 s at 95°C, and 1 minute at 60°C for 40 cycles. Expression levels were normalized to *GAPDH* (Hs02758991_g1), and relative gene expressions were assessed with ΔΔCt method.

### Immunohistochemical detection of COL-I

The samples were fixed in 4% buffered paraformaldehyde following decalcification in 14% EDTA solution. After equilibration in PBS buffered with 30% sucrose for 48 h, the samples were embedded in Tissue-Tek (O.C.T. compound, Sakura, CA, USA) and frozen at −80°C. 10 μm-thick sections (cryostat, Leica, Wetzlar, Germany) were mounted on Superfrost plus slides (Thermo Fisher Scientific). COL-I was detected using EnVision Detection Systems Peroxidase/DAB, Rabbit/Mouse (Dako/Agilent, Santa Clara, CA, USA) and anti-collagen I (Abcam, Cambridge, UK), as previously described (26). Human bone was used as a positive control. It was embedded in paraffin, followed by deparaffinization, and thus it was not designated for immunohistochemical comparison.

### Statistical analysis

The nature of the experiment (preparation of large number of high-concentration adenoviral vectors) was prohibitive of larger sample sizes, therefore the sample size was defined arbitrarily. Normality of the data distribution was tested by the Shapiro-Wilks test. Data are presented as a mean ± standard deviation (SD). Repeated measures ANOVA, one-way ANOVA, and *t* test were used for descriptive statistical analysis. *Post hoc* power analysis was performed for the DMP-1, for day 1 and 14 (T1 and T14), using repeated measurements method, with the following parameters: meanT1 1.11, SDT1 0.61; meanT14 80.62, SDT14 54.61. Entering these values to G*power software (*http://www.gpower.hhu.de/*) yielded the effect size of 1.46, which in turn yielded the overall β of 0.85. The level of significance was set at *P* < 0.050. Statistical analysis was performed using the TIBC Statistica version, 13.3 statistical model (TIBCO Software Inc. Palo Alto, CA, USA).

## RESULTS

### Phase 1: the optimal viral dose

**Figure 1:**
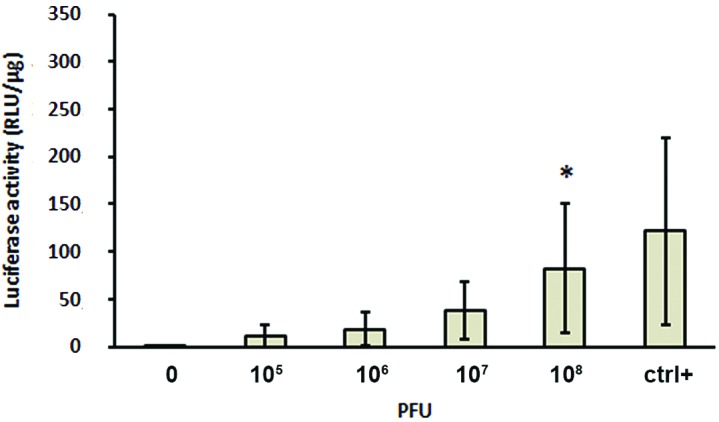
Human muscle tissue transduced with different doses of adenoviral vector carrying the luciferase reporter gene (Ad.Luc). The results are expressed as relative light units per microgram of protein (RLU/μg) and presented as mean ± standard deviation, n = 8, *P < 0.001. Positive control (ctrl+), human muscle tissue transduced with Ad.Luc infectivity 10^8^ plaque-forming units (PFU), was in contact with the virus for 72 h. Muscle tissue transduced for 2 hours with 0, 10^5^, 10^6^, 10^7^, and 10^8^ PFU differed significantly in luciferase activity. Muscle tissue transduced with Ad.Luc 10^8^ PFU showed the highest luciferase activity (82.10 ± 68.33; *P* < 0.001).

The results are shown as relative light units per microgram of protein (RLU/μg). Muscle tissue not transduced with Ad.Luc (negative control) did not show any luciferase activity. The positive control, muscle tissue transduced with the concentration of 10^8^ PFU and incubated to protein isolation 72 h later, showed the highest luciferase activity (mean 121.97 ± 98.55; *P* < 0.001 compared with negative control). Muscle tissue transduced with 10^5^, 10^6^, 10^7^, and 10^8^ PFU and incubated for 2 hours differed significantly in luciferase activity. Since 10^8^ PFU induced the highest luciferase activity compared with muscle tissue transduced with different PFU and incubated for 2 hours (mean 82.10 ± 68.33; *P* < 0.001), it was accepted as the optimal viral dose (Figure 1).

### Phase 2: the optimal contact time

**Figure 2:**
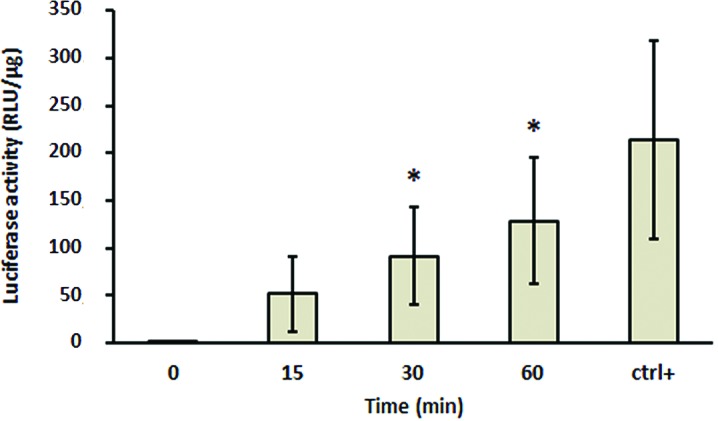
Human muscle tissue transduced with Ad.Luc 10^8^ under different contact times. The results are expressed as relative light units per microgram of protein (RLU/μg) and presented as mean ± standard deviation, n = 10, *P < 0.001. Positive control (ctrl+), human muscle tissue transduced with Ad.Luc infectivity 10^8^ plaque-forming units (PFU), was in contact with the virus for 72 h. Muscle tissue transduced with the Ad.Luc 10^8^ PFU and incubated for 30 and 60 min showed the highest luciferase activity.

Negative control did not show any luciferase activity, while the positive control showed the highest luciferase activity (mean 213.3 ± 104.18, *P* < 0.001 compared with the negative control). Muscle tissue transduced with the optimal dose of Ad.Luc and incubated for 15 min showed lower luciferase activity than all other samples and the positive control, and higher luciferase activity than the negative control (52.20 ± 39.59, *P* < 0.001). The tissue incubated for 30 min showed higher luciferase activity than the negative control and the tissue incubated for 15 min (91.73 ± 51.67, *P* < 0.001) but did not differ significantly from the tissue incubated for 60 min (128.21 ± 66.39; *P* = 0.187) (Figure 2). Therefore, the 30-min transduction was accepted as the optimal contact time.

### Phase 3: the effect of lanthanum and calcium ions on transduction

**Figure 3:**
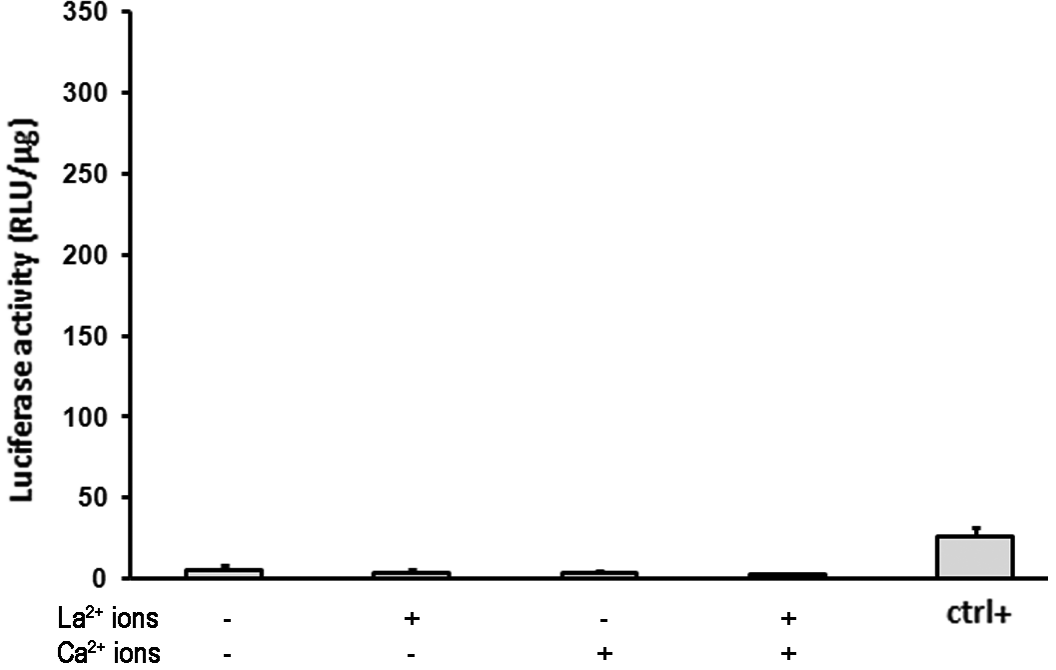
The effect of the addition of positive lanthanum ions (La^3+^) and calcium ions (Ca^2+^) on transduction enhancement. The results are expressed as relative light units per microgram of protein (RLU/μg) and presented as mean ± standard deviation, n = 10. Positive control (ctrl+), human muscle tissue transduced with Ad.Luc infectivity 10^8^ plaque-forming units (PFU), was in contact with the virus for 72 h. The presence of La^3+^ and Ca^2+^ did not improve the transduction.

Muscle tissue transduced with Ad.Luc infectivity 10^8^ PFU and incubated for 72 h until protein isolation (positive control) showed higher luciferase activity than all other samples (mean 25.80 ± 5.82, *P* < 0.001). Since the rest of the samples did not show significant luciferase activity, we concluded that La^3+^ and Ca^2+^ ions did not improve transduction (Figure 3).

### Phase 4: the application of optimized transduction protocols for enhanced osteodifferentiation of muscle tissue using AD.BMP-2

**Figure 4:**
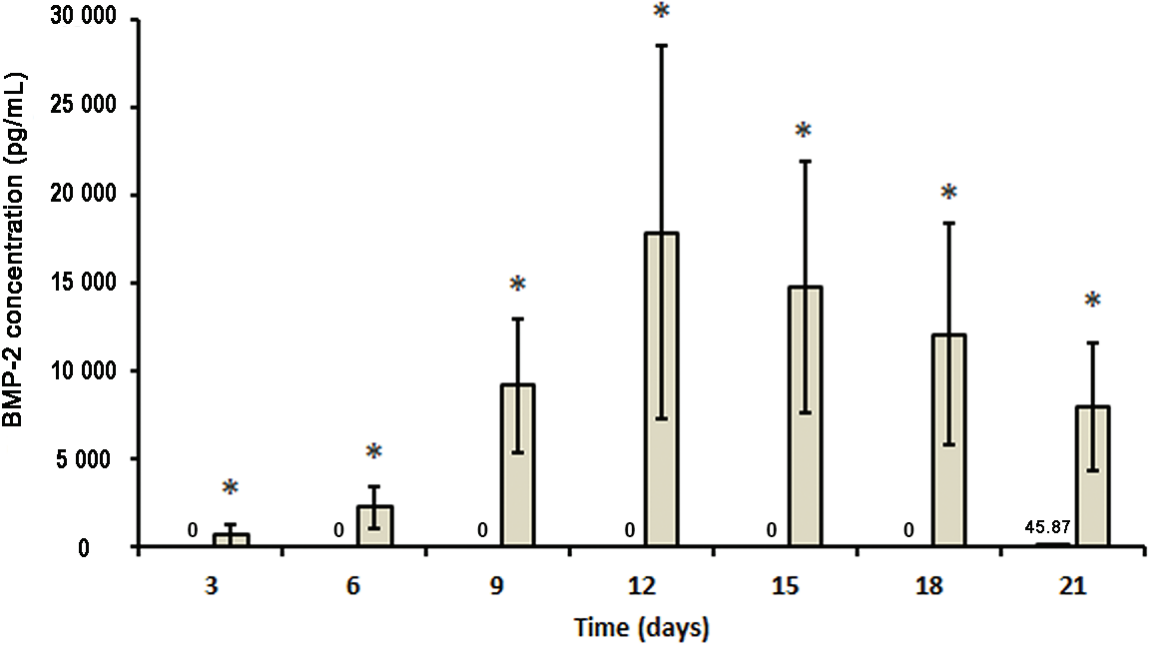
Expression of bone morphogenetic protein 2 (BMP-2) in native human muscle samples (black) and human muscle samples transduced with adenoviral vector carrying *BMP-2* gene (Ad.BMP-2) (gray). BMP-2 levels in osteogenic medium were measured every 3 days during 21 days by enzyme-linked immunosorbent assay. The results are presented as mean ± standard deviation (n = 3), *P < 0.001 for transduced muscle under the optimized protocol compared with the non-transduced muscle. Results of the muscle group are shown both graphically and numerically. Significantly higher *BMP-2* levels were observed in transduced muscle in comparison with non-transduced muscle. There was no difference in *BMP* expression between all transduced samples at all time points.

*Expression of BMP-2*. Transduced muscle showed significantly higher BMP-2 levels (*P* < 0.001) than non-transduced muscle. There was no difference in BMP-2 expression between all transduced samples at all time points. Looking at the absolute values, BMP-2 expression value in transduced muscles rapidly increased, peaked on day 12, and gradually decreased thereafter. Non-transduced muscle did not express BMP-2 until day 21, when low BMP-2 expression was observed (Figure 4).

**Figure 5:**
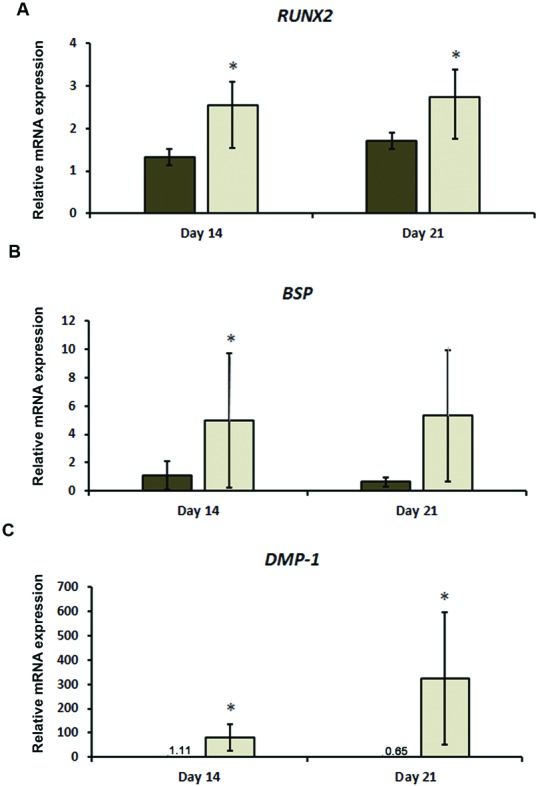
Expression of osteogenic differentiation markers in native human muscle samples (black) and human muscle samples transduced with adenoviral vector carrying *BMP-2* gene (Ad.BMP-2) (gray). Relative mRNA expression of (**A**) *runt-related transcription factor* (*RUNX2*), (**B**) *bone sialoprotein* (*BSP*), and (**C**) *dentin matrix protein 1* (*DMP-1*) was determined by real-time quantitative polymerase chain reaction on day 14 and 21. The results are presented as mean ± SD (n = 3), *P. Results of the muscle group in (**C**) are shown both graphically and numerically. The transduced muscle showed significantly higher *RUNX2* expression (*P* = 0.045) (**A**) and *DMP-1* gene expression than the non-transduced samples on days 14 and 21 (*P* < 0.001) (**C**). *BSP* expression was significantly higher on day 14 (*P* = 0.016) but not on day 21 (*P* = 0.059) (**B**).

*Expression of osteogenic markers*. Transduced muscle had significantly higher *RUNX2* gene expression (*P* = 0.045 on day 14 and day 21) (Figure 5A) and *DMP-1* gene expression (*P* < 0.001 on day 14 and day 21) (Figure 5C) than non-transduced muscle. *BSP* gene expression was significantly higher in transduced muscle than in non-transduced muscle on day 14 (*P* = 0.016) but not on day 21 (*P* = 0.059) (Figure 5B).

**Figure 6:**
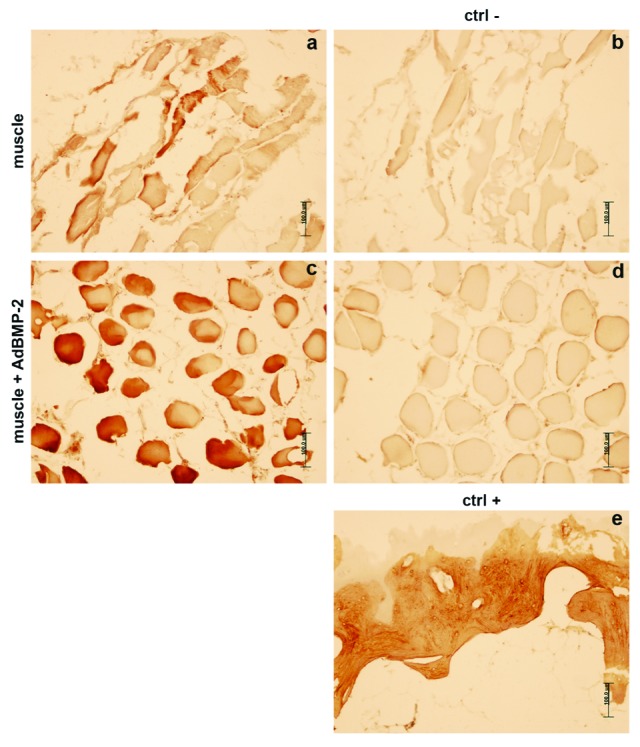
Immunohistochemical staining for collagen type I in muscle tissue transduced with Ad.BMP-2 under the optimized protocol. Positive staining is represented by brown color. Muscle tissue kept in osteogenic medium for 21 days was moderately stained (**A**). Transduced muscle tissue after 21 days of culture conditions was very strongly stained (**C**). Negative controls (-ctrl) were processed in the absence of suitable primary antibody (**B**),(**D**). Positive control was human bone (+ctrl) (**E**). Scale bar: 100 μm.

*Immunohistochemical detection of COL-I*. After 21 days of cultivation in osteogenic medium, non-transduced tissue showed low COL-I expression, while transduced muscle tissue showed strong COL-I expression (Figure 6).

## DISCUSSION

The results suggest that it is possible to develop a pioneer *ex vivo* gene therapy of human muscle tissue within 30 minutes by using Ad.BMP-2 to induce osteogenic differentiation. Translation of such strategies into clinical practice could enhance bone-tendon healing and prevent bone tunnel enlargement after ACL reconstruction.

During the ACL reconstruction, when the tendons are prepared for graft at the muscle end, the remaining part of the muscle tissue is removed and discarded. This part, however, could be preserved and used as a source of mesenchymal stem cells to stimulate tissue healing. Such a tissue could be used without any additional need for a donor site, thus reducing the potential donor site morbidity (ie, after obtaining bone marrow derived stem cells).

Adenoviral vectors have several advantages: they can be produced in a high titer, are highly infectious and able to infect cells in the resting and division phase, and their gene expression is transient due to episomal binding (27,28). Our results indicate that the highest dose of adenoviral vectors provides the best effect. Earlier studies also showed that the highest dose of adenoviral vectors improved transduction when bone defects were repaired using genetically modified muscles (18). Two critical limitations affecting the process are low infection efficiency at low doses and cytotoxicity at high doses, not only due to viral infection but also due to transgene expression. We used high viral doses, and if even higher doses are used, cytotoxicity could overpower the efficacy (29). As mentioned before, the nature of the experiment (preparations of large amounts of high concentration adenoviral vectors) was prohibitive of larger sample sizes.

Cell viability was not determined by MTT because cell abundance measurements, as MTT assay, work only if cell activity is constant, and cell activity can only be measured meaningfully if cells get the same MTT dose. Muscle grafts have a more complex cellular structure than a cellular monolayer, which reduces the reproducibility of MTT assay.

Samples of human muscle tissue incubated for 72 h showed the highest activity, but such a protocol is not acceptable for the expedited *ex vivo* gene therapy within a period shorter than two hours. By comparing the results of the shorter transduction periods, we accepted 30 minutes as the optimal time. The results obtained for this time period did not significantly differ from the longer transduction time (60 minutes) and were significantly higher compared with the shorter transduction time (15 minutes). The usual duration of orthopedic surgeries that need bone regeneration enhancement is from 60 minutes to several hours, so the use of gene therapy would not prolong the whole surgery, and thus would not increase the risk of complications associated with longer operating hours (infections, anesthesia complications, etc).

The addition of positive La^3+ ^and Ca^2+^ ions did not improve transduction, probably because their effect was disabled by high dose of adenoviral vector. For viral particles and ions, it is more difficult to penetrate tissues than to penetrate monolayer cells. Phosphate precipitates with adenoviruses can reach the cells located on the surface of treated tissue, but the proportion of these cells is too small to cause an overall viral infection. In addition, effects of La^3+^ are particularly strong at low viral doses (100 viral particles or ~ 1 PFU per cell). However, when a high dose of 50 000 viral particles per cell is used, the effect is decreasing (21). Since our viral dose was 10^8^ PFU, it is reasonable to conclude that the high viral dose decreased La^3+^ /Ca^2+^ influence.

The absolute values of the BMP expression rapidly increased in transduced muscles, peaked on day 12, and gradually decreased thereafter. The bone healing process lasts several weeks, and biological factors such as BMP-2 do not have to be permanently expressed. Future studies should monitor the duration of the episomal transgene expression, determine the time of decline in the quantity of expressed BMP-2, and assess which length and extent of BMP-2 expression is required for successful bone-formation. Earlier research on immunosuppressed Fischer rats found that a bone defect rapidly healed when autologous muscle tissue transduced with Ad.BMP-2 was used, although BMP-2 production was only in nanograms and lasted 1 to 3 weeks (30). The transplanted graft’s mechanical strength after ACL reconstruction within the bone tunnel is lowest around the sixth week of the postoperative period, which is a specificity of healing in the bone tunnel (31). It is important that biological signals that stimulate osteogenesis persist during this period, so the actual clinical effect of our method needs to be confirmed by further research. Moreover, further research should examine the osteogenic potential of the simultaneous use of two or more molecules on muscle cells (for example basic fibroblast growth factor and BMP-2 on bone marrow-derived stem cells) (32).

*RUNX2* regulates osteogenic development (33) and osteoblast differentiation (34,35), and induces the expression of osteogenic genes during osteoblast maturation (36). It is the first transcription factor needed to determine osteoblast presence (33). In our study, *RUNX2* was expressed both in non-transduced and transduced muscles, however its expression in transduced muscle was significantly higher at both measurement points. No difference in the expression was observed between days 14 and 21. The reason could be a sufficient long-lasting effect of BMP-2 on osteoprogenitor cells and their orientation toward immature osteoblasts throughout all three weeks. A study on genetically transduced rat muscle tissue using Ad.BMP-2 also showed significant *RUNX2* expression during 20 days (18).

*BSP* is a marker of osteoblast differentiation (37). In our study, gene expression of *BSP* marker was present in transduced and non-transduced muscles throughout the monitoring period. The BSP expression in transduced muscle on day 14 was significantly increased compared with the expression in non-transduced muscles. Increased *BSP* expression in transduced muscles indicates osteoblast maturation and orientation of muscle tissue toward the bone tissue production.

*DMP1* is an extracellular matrix protein important for proper bone mineralization (38,39). Although all tested samples expressed *DMP1*, transduced specimens showed significantly higher *DMP1* expression than non-transduced specimens. Also, transduced muscles showed 4 times higher expression on day 21 compared to day 14. The result suggests that a period of 3 weeks is sufficient for a successful transition from the osteoprogenitor to the osteocyte phase following the optimal transduction protocol of human muscle tissue transduced with recombinant adenoviral vector BMP-2. In this study, all human muscle tissue specimens were kept in an osteogenic differentiation medium and showed basal expression of osteogenesis markers. This result confirms osteogenic differentiation potential of standard osteogenic differentiation medium on fresh human muscle after *in vitro* cultivation (24).

As expected, non-transduced muscles expressed COL-I due to basal osteodifferentiation of muscle tissue in the osteogenic medium, but the expression was significantly higher in transduced muscles, which corresponds to intensified expression of osteogenic markers in these tissues.

The main limitations of this preclinical proof-of-concept study are the small number of patients used per group, insufficient statistical power to detect statistical significance for some findings, and the fact that the study was performed in controlled laboratory conditions without considering the interactions and mechanical forces within a living organism. For this study to be translated into clinical practice, additional well-designed studies and clinical trials should be carried out, as gene therapy is still viewed as unsafe and risky. Further studies will require close collaboration between scientists and clinicians.

In conclusion, we accelerated the process of *ex vivo* BMP-2 gene transfer to the hamstrings tendons muscle remnants, which facilitates its clinical application for improving intra-osseous bone-tendon healing after the ACL reconstruction. The 30-minute transduction protocol should be verified in *in vivo* research to bring it closer to clinical practice.

## References

[1] Ivkovic A, Marijanovic I, Hudetz D, Porter RM, Pecina M, Evans CH (2011). Regenerative medicine and tissue engineering in orthopaedic surgery.. Front Biosci (Elite Ed).

[2] Bruder SP, Fox BS (1999). Tissue engineering of bone: cell based strategies.. Clin Orthop Relat Res.

[3] Albrektsson T, Johansson C (2001). Osteoinduction, osteoconduction and osseointegration.. Eur Spine J.

[4] Virk MS, Sugiyama O, Park SH, Gambhir SS, Adams DJ, Drissi H (2011). “Same day” ex-vivo regional gene therapy: a novel strategy to enhance bone repair.. Mol Ther.

[5] Kawakami Y, Takayama K, Matsumoto T, Tang Y, Wang B, Mifune Y (2017). Anterior cruciate ligament–derived stem cells transduced with BMP2 accelerate graft-bone integration after ACL reconstruction.. Am J Sports Med.

[6] Vanden Bossche L, Vanderstraeten G (2005). Heterotopic ossification: a review.. J Rehabil Med.

[7] Kaplan FS, Le Merrer M, Glaser DL, Pignolo RJ, Goldsby RE, Kitterman JA (2008). Fibrodysplasia ossificans progressiva.. Best Pract Res Clin Rheumatol.

[8] Matthews BG, Torreggiani E, Roeder E, Matic I, Grcevic D, Kalajzic I (2016). Osteogenic potential of alpha smooth muscle actin expressing muscle resident progenitor cells.. Bone.

[9] Lee JY, Peng H, Usas A, Musgrave D, Cummins J, Pelinkovic D (2002). Enhancement of bone healing based on ex vivo gene therapy using human muscle-derived cells expressing bone morphogenetic protein 2.. Hum Gene Ther.

[10] Carreira ACO, Zambuzzi WF, Rossi MC, Astorino Filho R, Sogayar MC, Granjeiro JM (2015). Bone morphogenetic proteins: promising molecules for bone healing, bioengineering, and regenerative medicine.. Vitam Horm.

[11] Rahman MS, Akhtar N, Jamil HM, Banik RS, Asaduzzaman SM (2015). TGF-β/BMP signaling and other molecular events: regulation of osteoblastogenesis and bone formation.. Bone Res.

[12] Scarfi S (2016). Use of bone morphogenetic proteins in mesenchymal stem cell stimulation of cartilage and bone repair.. World J Stem Cells.

[13] Verma IM, Somia N (1997). Gene therapy-promises, problems and prospects.. Nature.

[14] Evans CH (2012). Gene delivery to bone.. Adv Drug Deliv Rev.

[15] Evans CH, Huard J (2015). Gene therapy approaches to regenerating the musculoskeletal system.. Nat Rev Rheumatol.

[16] Pelled G, Ben-Arav A, Hock C, Reynolds DG, Yazici C, Zilberman Y (2010). Direct gene therapy for bone regeneration: gene delivery, animal models, and outcome measures.. Tissue Eng Part B Rev.

[17] Rosenberg SA, Aebersold P, Cornetta K, Kasid A, Morgan RA, Moen R (1990). Gene transfer into humans—immunotherapy of patients with advanced melanoma, using tumor-infiltrating lymphocytes modified by retroviral gene transduction.. N Engl J Med.

[18] Evans CH, Liu F-J, Glatt V, Hoyland J, Kirker-Head C, Walsh A (2009). Use of genetically modified muscle and fat grafts to repair defects in bone and cartilage.. Eur Cell Mater.

[19] Arcasoy S, Latoche J, Gondor M, Pitt B, Pilewski J (1997). Polycations increase the efficiency of adenovirus-mediated gene transfer to epithelial and endothelial cells in vitro.. Gene Ther.

[20] Toyoda K, Andresen J, Zabner J, Faraci F, Heistad D (2000). Calcium phosphate precipitates augment adenovirus-mediated gene transfer to blood vessels in vitro and in vivo.. Gene Ther.

[21] Palmer G, Stoddart M, Gouze E, Gouze J, Ghivizzani S, Porter R (2008). A simple, lanthanide-based method to enhance the transduction efficiency of adenovirus vectors.. Gene Ther.

[22] Nilsson M, Ljungberg J, Richter J, Kiefer T, Magnusson M, Lieber A (2004). Development of an adenoviral vector system with adenovirus serotype 35 tropism; efficient transient gene transfer into primary malignant hematopoietic cells.. J Gene Med.

[23] Mizuguchi H, Hayakawa T (2004). Targeted adenovirus vectors.. Hum Gene Ther.

[24] Miao C, Zhou L, Tian L, Zhang Y, Zhang W, Yang F (2017). Osteogenic differentiation capacity of in vitro cultured human skeletal muscle for expedited bone tissue engineering.. BioMed Res Int.

[25] Ambriović Ristov A, Brozović A, Bruvo Mađarić B, editors. Methods in molecular biology. 1st ed. Zagreb: Institut Ruđer Bošković; 2007.

[26] Rogina A, Antunović M, Pribolšan L, Caput Mihalić K, Vukasović A, Ivković A (2017). human mesenchymal stem cells differentiation regulated by hydroxyapatite content within chitosan-based scaffolds under perfusion conditions.. Polymers (Basel).

[27] Ehrhardt A, Haase R, Schepers A, Deutsch MJ, Lipps HJ, Baiker A (2008). Episomal vectors for gene therapy.. Curr Gene Ther.

[28] Lee CS, Bishop ES, Zhang R, Yu X, Farina EM, Yan S (2017). Adenovirus-mediated gene delivery: Potential applications for gene and cell-based therapies in the new era of personalized medicine.. Genes Dis.

[29] Thomas CE, Birkett D, Anozie I, Castro MG, Lowenstein PR (2001). Acute direct adenoviral vector cytotoxicity and chronic, but not acute, inflammatory responses correlate with decreased vector-mediated transgene expression in the brain.. Mol Ther.

[30] Liu F, Ferreira E, Porter R, Glatt V, Schinhan M, Shen Z (2015). Rapid and reliable healing of critical size bone defects with genetically modified sheep muscle.. Eur Cell Mater.

[31] Tomita F, Yasuda K, Mikami S, Sakai T, Yamazaki S, Tohyama H (2001). Comparisons of intraosseous graft healing between the doubled flexor tendon graft and the bone–patellar tendon–bone graft in anterior cruciate ligament reconstruction.. Arthroscopy.

[32] Chen B, Li B, Qi YJ, Ni QB, Pan ZQ, Wang H (2016). Enhancement of tendon-to-bone healing after anterior cruciate ligament reconstruction using bone marrow-derived mesenchymal stem cells genetically modified with bFGF/BMP2.. Sci Rep.

[33] Komori T (2010). Regulation of osteoblast differentiation by Runx2.. Adv Exp Med Biol.

[34] Komori T (2006). Regulation of osteoblast differentiation by transcription factors.. J Cell Biochem.

[35] Hill TP, Später D, Taketo MM, Birchmeier W, Hartmann C (2005). Canonical Wnt/β-catenin signaling prevents osteoblasts from differentiating into chondrocytes.. Dev Cell.

[36] Cohen MM (2009). Perspectives on RUNX genes: an update.. Am J Med Genet A.

[37] Malaval L, Wade-Guéye NM, Boudiffa M, Fei J, Zirngibl R, Chen F (2008). Bone sialoprotein plays a functional role in bone formation and osteoclastogenesis.. J Exp Med.

[38] Narayanan K, Ramachandran A, Hao J, He G, Park KW, Cho M (2003). Dual functional roles of dentin matrix protein 1 Implications in biomineralization and gene transcription by activation of intracellular Ca2+ store.. J Biol Chem.

[39] Feng J, Huang H, Lu Y, Ye L, Xie Y, Tsutsui T (2003). The Dentin matrix protein 1 (Dmp1) is specifically expressed in mineralized, but not soft, tissues during development.. J Dent Res.

